# Oxidation of 5-Chloromethylfurfural (CMF) to 2,5-Diformylfuran (DFF)

**DOI:** 10.3390/molecules22020329

**Published:** 2017-02-20

**Authors:** Ana I. Vicente, Jaime A. S. Coelho, Svilen P. Simeonov, Hristina I. Lazarova, Margarita D. Popova, Carlos A. M. Afonso

**Affiliations:** 1 Research Institute for Medicines (iMed.ULisboa), Faculty of Pharmacy, Universidade de Lisboa, Av. Prof. Gama Pinto, 1649-003 Lisboa, Portugal; ana_vicente@netc.pt (A.I.V.); jaimeacoelho@gmail.com (J.A.S.C.); svilen.p.simeonov@gmail.com (S.P.S.); 2 Institute of Organic Chemistry with Centre of Phytochemistry Bulgarian Academy of Sciences, Acad. G. Bonchev str., bl. 9, 1113, Sofia, Bulgaria; lazarova@orgchm.bas.bg (H.I.L.); mpopova@orgchm.bas.bg (M.D.P.)

**Keywords:** 5-chloromethylfurfural, 2,5-diformylfuran, 5-hydroxymethylfurfural, oxidation, heterogeneous catalysis, catalyst reuse, flow chemistry

## Abstract

2,5-Diformylfuran (DFF) is an important biorenewable building block, namely for the manufacture of new polymers that may replace existing materials derived from limited fossil fuel resources. The current reported methods for the preparation of DFF are mainly derived from the oxidation of 5-hydroxymethylfurfural (HMF) and, to a lesser extent, directly from fructose. 5-Chloromethylfurfural (CMF) has been considered an alternative to HMF as an intermediate building block due to its advantages regarding stability, polarity, and availability from glucose and cellulose. The only reported method for the transformation of CMF to DFF is restricted to the use of DMSO as the solvent and oxidant. We envisioned that the transformation could be performed using more attractive conditions. To that end, we explored the oxidation of CMF to DFF by screening several oxidants such as H_2_O_2_, oxone, and pyridine *N*-oxide (PNO); different heating methods, namely thermal and microwave irradiation (MWI); and also flow conditions. The combination of PNO (4 equiv.) and Cu(OTf)_2_ (0.5 equiv.) in acetonitrile was identified as the best system, which lead to the formation of DFF in 54% yield under MWI for 5 min at 160 °C. Consequently, a range of different heterogeneous copper catalysts were tested, which allowed for catalyst reuse. Similar results were also observed under flow conditions using copper immobilized on silica under thermal heating at 160 °C for a residence time of 2.7 min. Finally, HMF and 5,5′-oxybis(5-methylene-2-furaldehyde) (OBMF) were the only byproducts identified under the reaction conditions studied.

## 1. Introduction

The pursuit of new biorenewable building blocks and/or more efficient synthetic routes has intensified during the last few years. To date, several molecules including ethanol, glycerol, carboxylic acids (lactic acid, succinic acid, 3-hydroxypropionic acid, itaconic acid, glutamic acid, levulinic acid, fatty acids), furfural, 5-hydroxymethylfurfural (HMF) and terpenes (α-pinene, limonene, citral, menthol) [1,2,3,4] have been identified as alternatives to petroleum-based building blocks. The discovery of new polymer monomers that may replace the current well established portfolio derived from fossil fuel resources has been recognized as an important issue. 2,5-Furandicarboxylic acid (FDCA), 2,5-dihydroxymethylfuran (DHMF) and 2,5-diformylfuran (DFF), among others, have been identified as promising monomers [5,6]. These furan derivatives are commonly obtained from HMF, which, in turn, is mainly derived from fructose [7,8,9,10,11]. Besides the intensive research and remarkable achievements regarding the production of HMF, there are still limitations that arise from the specific nature of HMF, namely, its thermal and chemical instability, high solubility in water, and low melting point. On the other hand, as highlighted by the Mascal group, 5-chloromethylfurfural (CMF) is an appealing alternative to HMF, due to its higher stability, lower polarity and higher accessibility from glucose and cellulose [12,13,14].

**Scheme 1:**
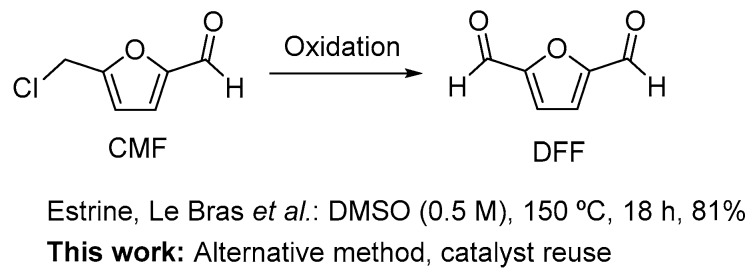
Reported methods by Estrine, Le Bras et al. [38] for the oxidation of CMF to DFF and in this study on the search for alternative methods that avoid the use of DMSO and allow catalyst reuse.

In general, DFF is prepared by the oxidation of HMF [8,11]. The selective oxidation of the hydroxyl group into the aldehyde has been achieved using a variety of different methodologies including homogeneous [8,11,15,16] and heterogeneous [8,11,17,18,19,20,21,22,23,24,25,26,27,28,29,30,31,32,33,34,35,36,37] metal catalysis, a one-step procedure from fructose (DMSO, NaBr (cat.), 150 °C, 50%) [38], using molybdovanadophosphoric heteropolyacids [39,40], using polyaniline-grafted vanadyl acetylacetonate [41], metal free procedures [42,43,44] and enzyme catalysis [45]. The use of CMF as a starting material for the preparation of DFF was previously described in 2014 by Estrine and Le Bras and later used by the Mascal group. However, the use of DMSO as the solvent and oxidant makes this method less attractive (Scheme 1) [38,46]. To that end, we envisioned the possibility of performing such an oxidation without using DMSO (Scheme 1).

## 2. Results and Discussion

The oxidation of benzylic chlorides to the corresponding aldehydes has been reported using different systems, namely CuCl/Kieselguhr/O_2_ [47], 2-dimethylamino-*N*,*N*-dimethylaniline *N*-oxide [48], 3,6-bis(triphenylphosphonium)cyclohexene peroxodisulfate [49], NaIO_4_-DMF [50], H_5_IO_6_ in [C_12_mim][FeCl_4_] ionic liquid [51], TEMPO (cat)/KNO_2_ (cat)/O_2_ [52], NMO/KI (cat) in [emim]Cl ionic liquid, MW [53] and molybdate-based catalyst/H_2_O_2_ [54]. Based on that, we started this study by screening several oxidants (1.5 equiv.) for the oxidation of CMF in tetrabutylammonoium chloride (TBAC) as a reaction medium under basic conditions (K_2_CO_3_, 2 equiv.) at 110 °C for 5 h. Several oxidants such as urea hydrogen peroxide (UHP), NaBO_3_, Ca(ClO)_2_, NaIO_4_, H_2_O_2_, Oxone, KNO_2_, and NMO yielded full conversion of CMF. However, very low NMR yields of DFF were obtained (<10%, Table S1). In addition, HMF was identified as the single by-product (yields ranging from 3% to 67%), whose formation may derive from the nucleophilic substitution of the chloride atom. Those unsatisfactory results suggest that TBAC salt is not suitable for this transformation under the tested reaction conditions, giving side reactions such as over oxidation and nucleophilic substitution.

From the preliminary screening, pyridine *N-*oxide (PNO) in combination with CuCl (0.5 equiv.) was identified as the most effective system, providing DFF in 12% yield. Further solvent screening using PNO (2 equiv.) as the oxidant at lower temperature (82 °C) for 24 h produced DFF in up to 9% yield. (Table 1, entries 1–5). Acetonitrile was identified as the most promising solvent, suppressing the undesired formation of HMF (Table 1, entry 1). Furthermore, no conversion of CMF was observed in the absence of the promoter CuCl (Table 1, entry 4). Increasing the amount of PNO to 4 equiv. resulted in an increased yield of DFF to 37% (3% of unreacted CMF was detected). However, HMF was detected in 26% yield (Table 1, entry 6). The use of both anhydrous acetonitrile and PNO was not beneficial for the suppression of HMF formation probably due to trace amounts of water in the PNO (Table 1, entry 7). The use of longer reaction times (55 h, 34%, Table 1, entry 8), higher excess of PNO (8 equiv., 37% yield, Table 1, entry 9), or lower/higher temperatures (60 °C: 13%; 110 °C: 19%; Table 1, entries 10 and 11) were not beneficial for the transformation. In addition, Cu(OTf)_2_ provided comparable results (35%; Table 1, entry 12). Interestingly, using HMF instead of CMF resulted in no formation of DFF, which suggests that DFF is not formed via HMF oxidation (Table 1, entry 14).

The use of microwave irradiation (MWI) using 4 equiv. of PNO and 0.5 equiv. of Cu(OTf)_2_ in acetonitrile (0.5 M) at 82 °C lead to the formation of DFF in 49% yield after 6 h, together with 24% of unreacted CMF (Table 2, entry 1). Remarkably, the formation of HMF was not observed under these conditions, which is considerably better than the best results achieved under thermal heating (37% of DFF after 24 h, Table 1, entry 6). Further optimization of the reaction conditions to 160 °C and only 5 min of reaction time resulted in the formation of DFF in up to 51% yield (isolated yield of 50%, Table 2, entries 9 and 10, see complete data in Table S3). In addition, the reaction performed in the absence of the promoter Cu(OTf)_2_ yielded only 6% of DFF (Table 2, entry 11). Finally, no formation of DFF was observed in the absence of the PNO oxidant, resulting in the complete decomposition of the CMF substrate instead (Table 2, entry 15).

**Figure 1:**
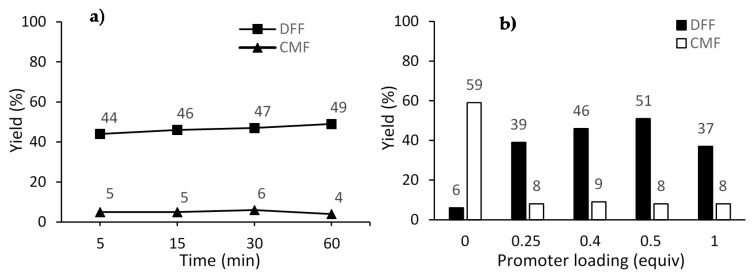
Oxidation of CMF for (**a**) different reaction times at 140 °C using 0.5 equiv. of Cu(OTf)_2_; (**b**) different promoter loading at 160 °C for 5 min. *Reaction conditions*: microwave irradiation (MWI), CMF (0.35 mmol, 50 mg), 4 equiv. of pyridine *N*-oxide, in CH_3_CN (0.5 M). Yields were determined by ^1^H-NMR analysis of the crude reaction mixture using 1,3,5-trimethoxybenzene as internal standard.

The study of the reaction time was carried out following the general procedure at 140 °C with 0.5 equiv. of Cu(OTf)_2_. The results shown in Figure 1a indicate that there is no pronounced advantage for both the DFF yield and CMF conversion by running the reaction for more than 5 min. Regarding the promoter loading, it was observed that 0.5 equiv. of Cu(OTf)_2_ is the optimal amount, yielding the product DFF in 51% yield.

**Figure 2:**
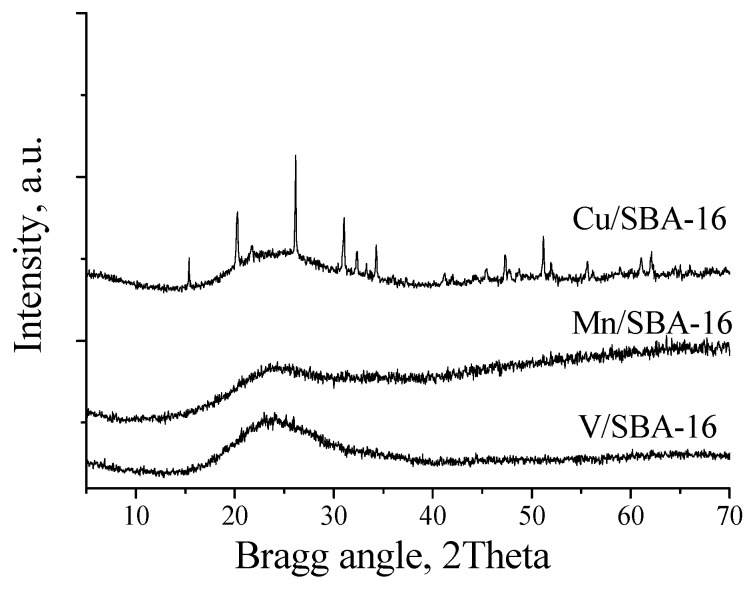
XRD temperature-programmed reduction of the Cu/SBA-16, V/SBA-16 and Mn/SBA-16 catalysts.

We envisioned that this procedure would be more effective under heterogeneous conditions. Based on that, several different catalysts, namely commercially available CuCl or CuSO_4_ as well as supported Cu catalysts, were tested under the conditions optimized using the promoter Cu(OTf)_2_ (0.5 mol %, PNO (4 equiv.), acetonitrile (0.5 M), MWI, 160 °C, 5 min). The adsorption of CuSO_4_ in silica (CuSO_4_·Si) and neutral (CuSO_4_·AN) or basic alumina (CuSO_4_·AB) was performed by simple evaporation of the solid support suspension in an aqueous solution of CuSO_4_. TheCu/SiO_2_ catalysts were prepared according to the reported procedure [55] by dispersion of the copper complex in silica followed by water evaporation and calcination (400 °C, 4 h). The SBA-16 catalysts were synthesized according to the procedure of Hu et al. [56]. The XRD data of the Cu, Mn, and V modified SBA-16 silica materials in the lower two theta regions (not shown) confirm the preservation of the mesoporous structure after the impregnation process. In the higher two theta regions, reflections with low intensity, characteristic of CuO, were detected in the Cu/SBA-16 material. No reflections typical of Mn and V oxide species could be observed by XRD (Figure 2), which is due to the formation of finely dispersed metal oxide species in them. The crystallite size of the copper oxide particles was determined by the Scherrer method to be around 20 nm. The calculated textural parameters from the nitrogen physisorption measurements of the metal-containing silica materials are listed in Table 3. No significant pore size reduction can be observed in SBA-16 supported catalysts either in the spherical pores (~8 nm) or in the interconnecting channels (2.5 nm). These data support that a portion of the metal oxides penetrated into the channels and cavities of the mesoporous supports, but no significant pore blocking occurred.

**Figure 3:**
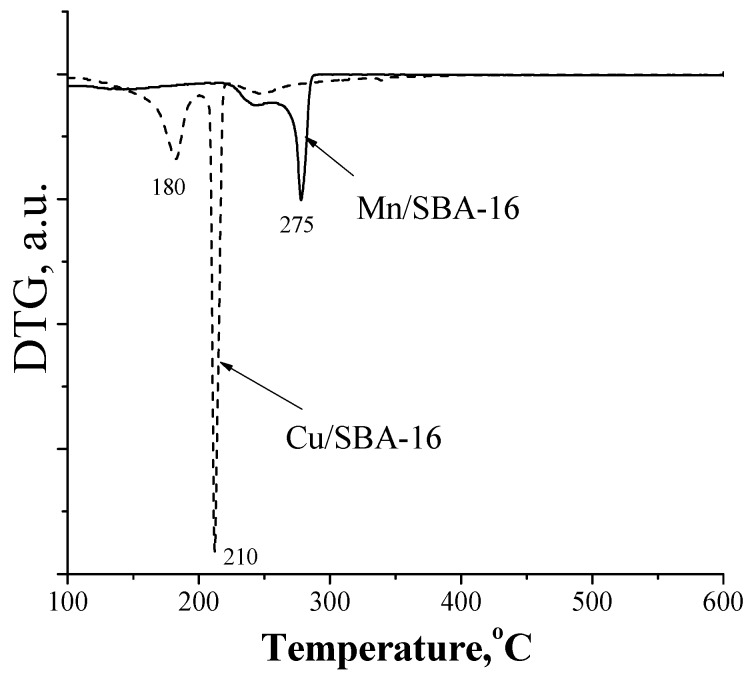
TPR of the Cu/SBA-16 and Mn/SBA-16 catalysts.

The temperature-programmed reduction (TPR) data (Figure 3) show the total reduction of CuO to Cu(o) in the Cu/SBA-16 sample which occurred at low temperature (around 210 °C). Partial reduction of the Mn oxide species was detected in the Mn/SBA-16 sample at higher temperature (275 °C).

**Figure 4:**
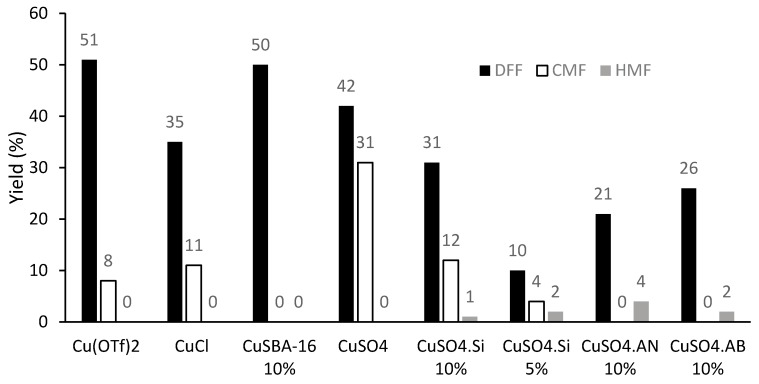
Effect of the promoter nature on the oxidation of CMF under microwave irradiation (MWI). *Reaction conditions*: CMF (0.35 mmol, 50 mg), catalyst, PNO (4 equiv.) in CH_3_CN (0.5 M) and 160 °C for 5 min under MWI; yields were determined by ^1^H-NMR analysis of the crude reaction mixture using 1,3,5-trimethoxybenzene as an internal standard.

Oxidation of CMF in the presence of different heterogeneous catalysts indicated that CuSBA-16 (10%) is the most efficient promoter, leading to a similar yield of DFF as the homogeneous promoter Cu(OTf)_2_. Remarkably, CuSO_4_, which is the cheapest catalyst tested, gave 42% yield of DFF (Figure 4). Using a higher amount of CuSO_4_ (1%), longer reaction time (up to 30 min), or higher temperature (up to 170 °C) gave lower yields of DFF (38%, 41%, and 35%, respectively), together with the formation of small amounts of HMF (up to 7%, see full results in Table S4).

**Figure 5:**
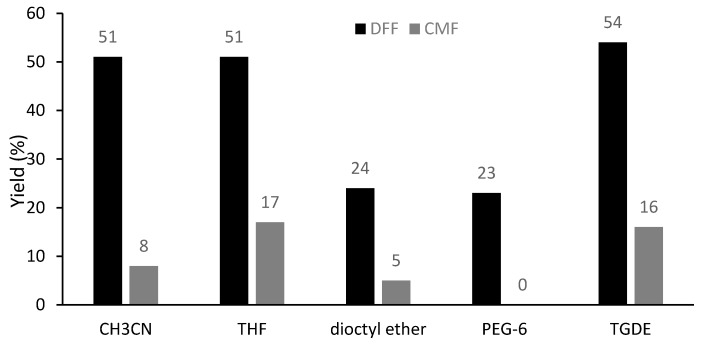
Effect of the solvent on the oxidation of CMF under microwave irradiation (MWI). *Reaction conditions*: CMF (0.35 mmol, 50 mg), Cu(OTf)_2_ (0.5 equiv.), PNO (4 equiv.) in a concentration of 0.5 M at 160 °C for 5 min under MWI; yields were determined by ^1^H-NMR analysis of the crude reaction mixture using 1,3,5-trimethoxybenzene as an internal standard.

In order to facilitate the flow process, several ethers were tested as solvents for the oxidation of CMF using Cu(OTf)_2_ as the promoter, including those with high boiling points (b.p. higher than 160 °C) such as dioctyl ether, PEG-6 and triethylene glycol dimethyl ether (TGDE) (Figure 5). Remarkably, TGDE gave a better yield of DFF (54%) than acetonitrile, together with 16% of unreacted CMF. In addition, the oxidation is also feasible using CuSO_4_ or supported CuSO_4_ on silica (CuSO_4_·Si (10%)) in TGDE, yielding DFF in 33% and 44%, respectively (Table S5).

**Figure 6:**
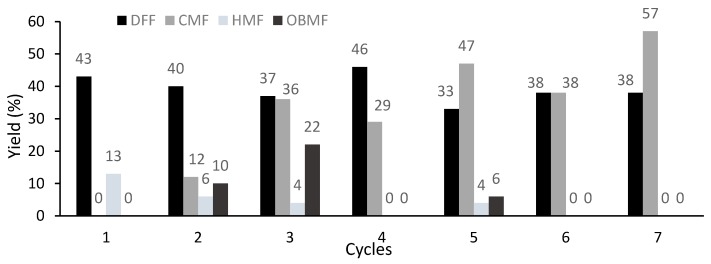
Catalyst reuse for the oxidation of CMF. *Reaction conditions for each cycle*: CMF (0.35 mmol, 50 mg), 0.5 equiv. of CuSO_4_·Si 10%, 4 equiv. of PNO in TGDE (0.5 M) at 160 °C for 5 min under microwave irradiation; yields were determined by ^1^H-NMR analysis of the crude reaction mixture using 1,3,5-trimethoxybenzene as an internal standard. OBMF: 5,5′-oxybis(5-methylene-2-furaldehyde).

The heterogeneous catalyst CuSO_4_·Si (10%) was selected to explore catalyst reuse (Figure 6). Remarkably, only a slight erosion of the DFF yield was observed over seven cycles. (1st cycle: 43%; 7th cycle: 38%). In addition, the amount of unreacted CMF increased from cycle to cycle (1st cycle: none; 7th cycle: 57%). Interestingly, for the cycles in which the formation of HMF was observed, formation of the dimer OBMF was also observed, which could arise from the reaction of HMF with CMF.

Finally, the oxidation of CMF was explored under flow conditions. Initially, the flow study started by testing the efficiency of the catalyst by performing four oxidation reactions in continuous flow conditions. The selected conditions to perform the flow experiments were based on the best conditions obtained under microwave irradiation and using TGDE (b.p. = 216 °C) as the solvent because it allows the reaction to be performed at 160 °C without causing pressure issues. The other reaction parameters used were: 0.5 mL/min flow rate and 10% (wt/wt) CuSO_4_ adsorbed on silica catalyst. The yields obtained for each identified product or starting material are presented in Table 4 for four consecutive flow experiments using the same catalyst. It is worth mentioning that in contrast to the results obtained under batch conditions (thermal and MWI), the mass balance is almost complete for all of the experiments, indicating that there are no side products besides the formation of HMF and OBMF. For the first reaction, the yields of DFF and CMF are similar to the ones obtained under MWI or batch conditions using the same solvent, temperature and catalyst, 43% vs. 44% and 15% vs. 22%, respectively (Table 4 vs. Table S5). Under flow conditions, the formation of HMF leading to an almost complete mass balance was observed, while under MWI conditions, the mass balance was only 66%, which suggests that the flow may minimize further transformations such as over oxidation. These polar products are probably absorbed on the silica when the reaction solution is filtered to separate the catalyst from the crude and this could explain the incomplete mass balance for the microwave reactions. Through the consecutive reactions, the yields of DFF, CMF and HMF decrease while the formation of a new product was observed in up to 77%—the dimer OBMF. These results indicate that decreasing the activity of the catalyst results in the formation of HMF that consequently leads to the formation of OBMF.

Since CMF was not totally consumed, we tried to improve the yield of DFF by using the same flow conditions as previously described, but at 180 °C. However, a lower performance was observed providing yields of 20%, 16%, 43% and 20% of DFF, CMF, HMF and OBMF, respectively, indicating that these conditions are detrimental for the formation of DFF (Table 4 in brackets).

In order to minimize the side reaction of HMF formation, which appeared to be promoted under flow conditions and was probably due to the presence of water, the catalyst was dried for 24 h at 130 °C. However, there was no improvement in the results (Table 5, entry 2 vs. 1). Since the TGDE solvent is highly hygroscopic, the solvent was changed to tetraethylene glycol dimethyl ether (TEGDME, b.p. = 275 °C), that is less hygroscopic, providing only a slightly higher yield of DFF (40% vs. 36% for TGDE, Table 5, entry 3 vs. 2) and only a 17% yield reduction of HMF, although with the formation of OBMF in a 10% yield. Milled hydrated CuSO_4_ was also tested, resulting in a lower yield of DFF (17%), together with a higher yield of HMF (71%). In addition, an increase in the pressure was observed, which was due to column blocking during operation. In order to circumvent this limitation, a lower amount (5%) of CuSO_4_ immobilized in silica was tested, resulting in comparable results to the ones observed for 10% CuSO_4_ in silica (Table 5, entry 5 vs. entry 1). A 1:1 mixture of Cu_2_O/silica gave only a 37% yield of DFF as the only detected product (Table 5, entry 6). The use of Cu/SiO_2_ prepared according to a reported procedure [55], gave a 29% yield of DFF and a 49% yield of undesired HMF, which was less efficient then CuSO_4_ supported in silica. In order to reduce subsequent over oxidation, lower residence times were tested (Table 5, entries 9–10) by increasing the flow rate from 0.5 to 1 mL/min. This condition allowed for the formation of DFF with 42% yield. In addition, increasing the amount of promoter to 1 equiv. gave a 54% yield of DFF (Table 5 entry 10). The CMF loading was also increased by passing 1 g through the column containing the immobilized catalyst Cu/SiO_2_ and all CMF was consumed, yielding 27% of DFF and 54% of HMF (Table S10).

## 3. Materials and Methods

### 3.1. General Materials and Methods

Tetrabutylammonium chloride (TBAC), 1,3,5-trimethoxybenzene, urea hydrogen peroxide (UHP), sodium perborate tetrahydrate (NaBO_3_·4H_2_O), calcium hypochlorite (Ca(ClO)_2_), sodium periodate (NaIO_4_), hydrogen peroxide (H_2_O_2_), oxone (2KHSO_5_·KHSO_4_·K_2_SO_4_), potassium nitrite (KNO_2_), *N*-methylmorpholine-*N*-oxide (NMO), potassium carbonate (K_2_CO_3_), potassium bromide (KBr), pyridine *N*-oxide (PNO), (2,2,6,6-tetramethylpiperidin-1-yl)oxyl (TEMPO), sodium nitrite (NaNO_2_), *p*-tolyl sulfoxide, chlorotrimethylsilane, copper(I) chloride (CuCl), copper(II) triflate (Cu(OTf)_2_), copper(I) triflate toluene complex (Cu(OTf)), copper oxide (Cu_2_O), and copper(II) sulphate (CuSO_4_) were purchased from Sigma-Aldrich (Lisboa, Portugal) and used as received unless otherwise stated. Column chromatography was performed using silica gel 60 GF254 Merck Ref. 1.07730.1000 (Darmstadt, Germany). Catalysts were adsorbed or immobilized in silica gel 60 GF254 MercK Ref. 1.07730.1000, aluminium oxide 90 active basic MercK Ref. 101076 (AB), or aluminium oxide 90 active neutral MercK Ref. 101077 (AN). The reagents dioctyl ether (Ref. 249599), tetraethylene glycol dimethyl ether (Ref. 172405) (TEGDME), and triethylene glycol dimethyl ether (Ref. 8.08249.0250) (TGDE) were purchased from Sigma Aldrich and polyglycol 300 (PEG-6) was obtained from Clariant (Artziniega, Spain). All solvents, tetrahydrofuran (THF), acetonitrile, *tert*-butyl alcohol (*t*-BuOH), chloroform, ethyl acetate, dichloromethane, and *n*-hexane were used without further purification.

5-(Hydroxymethyl)furfural (HMF) was synthetized from fructose using the procedure previously reported by our group [57,58]. 5-(chloromethyl)furan-2-carbaldehyde (CMF) was prepared from HMF following a reported procedure [59].

Nuclear magnetic resonance (NMR) spectra were recorded using a Bruker MX300 spectrometer (Rheinstetten, Germany). The microwave experiments were performed on a CEM Discover Benchmat apparatus (CEM Corporation, Matthews, NC, USA) using a glass pressure vessel (10 mL).

### 3.2. Preparation of Heterogeneous Catalysts

General procedure for the adsorption of CuSO_4_ in silica, basic alumina or neutral alumina is as follows: the desired solid support (10 g) was added to CuSO_4_ (1 g) in water (50 mL). After stirring for 10 min, water was removed under vacuum in a rotary evaporator and left in an oven at 130 °C for 24 h. The resulting solid was used without further treatment.

General procedure for the preparation of the Cu/SiO_2_ immobilized catalysts: the Cu/SiO_2_ catalysts were prepared according to a reported procedure [55]. Briefly, to prepare a catalyst with x%, NH_4_OH was added to a solution of Cu(NO_3_)_2_·2.5H_2_O until pH 9. The silica was added and stirred for 20 min. The solution was submerged in an ice bath at 0 °C and diluted with water in order to hydrolyze the copper complex and precipitate the dispersed product. The solid was filtered and washed with water, dried overnight at 110 °C and calcined at 400 °C for 4 h.

Synthesis of SBA-16 and its Mn-, Cu- and V-modified analogs: the parent silica SBA-16 material was synthesized according to the procedure of Hu et al. [56]. Pluronic F127 triblock copolymer and cetyl trimethyl ammonium bromide (CTAB) were used as templates and tetraethylortosilicate (TEOS) was used as a silica source. In a typical synthesis, F127 (1.0 g) and CTAB (0.12 g) were completely dissolved into a solution of 130 mL water and 10 mL concentrated HCl, followed by the addition of 4.0 mL of TEOS under stirring. After 1 h of stirring at 40 °C, the mixture was heated at 80 °C for 24 h under static conditions. The solid product was filtered, washed with water three times and dried at 50 °C. The template was removed by calcination in air at 550 °C for 5 h with a heating rate of 1 °C/min.

Mn, Cu, V-containing (5–20 wt %) SBA-16 materials were prepared by incipient wetness impregnation with manganese (II) acetylacetonate, copper (II) acetylacetonate and vanadyl(VI) sulphate as salt precursors, respectively.

Characterization of the initial and modified SBA-16 materials: the X-ray powder diffraction (XRD) patterns were recorded on a PANalytical X´Pert PRO (HTK) high-resolution diffractometer (The Netherlands) using Cu Kα1 radiation (1.5406 Å) in the 2 θ range from 5° to 80° (100 s per 0.016° step) for the samples and from 10° to 70° (100 s per 0.016° step) for the sample holder using a fully opened X´Celerator detector.

Nitrogen physisorption measurements were carried out at −196 °C using a Tristar 3000 Micromeritics volumetric adsorption analyzer (Ense, Germany). Before the adsorption analysis, the samples were outgassed under vacuum for 2 h at 200 °C in the port of the adsorption analyzer. The Brunauer–Emmett–Teller (BET) specific surface area was calculated from adsorption data in the relative pressure range from 0.05 to 0.21. The pore size distributions (PSDs) were calculated from the nitrogen adsorption data using an algorithm based on the ideas of Barrett, Joyner and Halenda (BJH).

The reducibility of the Cu, Mn, and V-modified samples were investigated by the temperature-programmed reduction (TPR) technique in H_2_/Ar flow (10:90, 20 mL/min) using a conventional TPR apparatus equipped with a heat conductivity cell and a trap for the removal of released water. Before TPR, run samples were pretreated in oxygen at 350 °C for 1 h.

General procedure for the oxidation of CMF under microwave irradiation (MWI): CMF (0.35 mmol, 50 mg), pyridine *N*-oxide (4 equiv., 1.4 mmol), Cu(OTf)_2_ (0.5 equiv.) and CH_3_CN (0.5 M) were added to a microwave pressure vessel (10 mL). The resulting mixture was allowed to stir at the desired temperature and time. The mixture was filtered through a pad of silica gel using a mixture of hexane/ethyl acetate (1:1) and the solvents were evaporated. Quantification of CMF, DFF, and HMF was performed by ^1^H-NMR analysis of the crude reaction mixture by using 1,3,5-trimethoxybenzene as an internal standard. DFF and CMF were isolated by column chromatography purification using hexane:ethyl acetate (2:1).

Procedure for the oxidation of CMF and catalyst reuse: CMF (0.35 mmol, 50 mg), pyridine *N*-oxide (4 equiv., 1.4 mmol), CuSO_4_·Si (10% *w*/*w*, 0.5 equiv.) and TGDE (0.5 M) was added to a microwave pressure vessel (10 mL). The resulting mixture was stirred for 5 min at 160 °C under microwave irradiation. The mixture was filtered first through cotton using a Pasteur pipette (to collect the catalyst) followed by filtration through silica using a mixture of hexane/ethyl acetate 1:1 and the solvents were removed under vacuum. Quantification of CMF, DFF, HMF and (5,5′-(oxybis(methylene))bis(furan-5,2-diyl))dimethanol (OBMF) was performed by ^1^H-NMR analysis of the crude mixture using 1,3,5-trimethoxybenzene as an internal standard. The collected catalyst was then reused in the next cycle using the same reaction conditions.

General procedure for CMF oxidation using flow conditions: an empty HPLC column (i.d. = 3.9 mm; L = 300 mm) was filled with the respective modified silica and/or promoter and placed inside a gas chromatography (GC) oven. The desired solvent was passed through the column at room temperature using an HPLC pump until the column was completely filled. The temperature of the GC oven was then raised to the desired value and stabilized for 30 min. A solution of CMF (1.56 mmol, 225 mg) and pyridine *N*-oxide (4 equiv.) in the respective solvent (0.5 M in CMF) was passed through the column under constant flow and the output was sequentially collected in different vials.

Note 1: Quantification by ^1^H-NMR was performed taking into account the weight of the collected fraction and by adding a specific amount of the internal standard (1,3,5-trimethoxybenzene).

Note 2: The promoter amount was calculated by weighing the column before and after filling with the modified silica. When necessary, the resulting empty volume of the column was filled with silica.

For the cases in which the column was blocked during the operation, the experiment was repeated by filling the end of the column with silica followed by the supported catalyst.

## 4. Conclusions

This study describes the oxidation of CMF to DFF by exploring a range of oxidants, promoters and reaction conditions. Pyridine *N*-oxide (PNO) in combination with the Cu based promoter under homogeneous and heterogeneous conditions was identified as the best system. In addition, the heterogeneous catalyst can be reused and applied under flow conditions providing similar performance as obtained under homogeneous conditions.

## Figures and Tables

**Table 1:** Optimization of reaction conditions for the oxidation of CMF using PNO as an oxidant under thermal heating.

Entry	Solvent	Promoter	Oxidant (equiv.)	T (°C)	Yield (%)			
					DFF	CMF	HMF	Total
1	CH_3_CN	CuCl	2	82	9	49	n.d.	58
2	*t*-BuOH	CuCl	2	82	5	47	48	100
3	THF	CuCl	2	82	6	n.d.	96	100
4	CH_3_CN	None	2	82	n.d.	100	n.d.	100
5	CH_3_CN:H_2_O 1:1	CuCl	2	82	n.d.	n.d.	100	100
6	CH_3_CN	CuCl	4	82	37	3	26	66
7	CH_3_CN ^a^	CuCl	4	82	25	3	53	81
8	CH_3_CN ^b^	CuCl	4	82	34	14	n.d.	48
9	CH_3_CN	CuCl	8	82	37	8	4	49
10	CH_3_CN	CuCl	4	60	13	2	45	60
11	CH_3_CN	CuCl	4	110	19	n.d.	n.d.	19
12	CH_3_CN	Cu(OTf)_2_	4	82	35	5	n.d.	40
13	CH_3_CN ^c^	Cu(OTf)_2_	4	82	32	2	4	38
14	CH_3_CN ^d^	CuCl	4	82	n.d.	100	n.d.	100

*Reaction conditions:* CMF (50 mg, 0.35 mmol), promoter (0.5 equiv.), pyridine *N*-oxide as oxidant, in a concentration of 0.5 M at 82 °C for 24 h in a glass pressure tube (Aldrich Ref. Z181064-1EA); the yields were determined by ^1^H-NMR using 1,3,5-trimethoxybenzene as an internal standard. ^a^ Using dry PNO and CH_3_CN; ^b^ reaction time is 55 h instead of 24 h; ^c^ using0.25 equiv. of promoter; ^d^ reaction performed using HMF (0.35 mmol) instead of CMF; n.d. = not detected.

**Table 2:** Selected reaction conditions and yields of DFF, CMF, and HMF for the oxidation reactions of CMF performed under microwave irradiation (MWI).

Entry	Time (min)	Temp. (°C)	Oxidant (equiv.)	Promoter (equiv.)	Yield (%)		
					DFF	CMF	Total
1	360	82	4	0.5	49	24	73
2	120	100	4	0.5	39	4	43
3	60	120	4	0.5	39	7	46
4	5	140	4	0.5	44	5	49
5	15	140	4	0.5	46	5	51
6	30	140	4	0.5	47	6	53
7	60	140	4	0.5	49	4	53
8	1	160	4	0.5	47	18	65
9	5	160	4	0.5	51	8	59
10	5 ^a^	160	4	0.5	50	8	58
11	5	160	4	0	6	59	65
12	5	160	4	0.25	39	8	47
13	5	160	4	1	37	8	45
14	5	160	2	0.5	40	n.d.	40
15	5	160	0	0.5	dark crude		
16	30	160	4	0.5	45	4	49
17	1	170	4	0.5	49	3	52

*Reaction conditions*: CMF (0.35 mmol, 50 mg), Cu(OTf)_2_, pyridine *N*-oxide as oxidant, in CH_3_CN (0.5 M) under microwave irradiation (MWI); the yields were determined by ^1^H-NMR using 1,3,5-trimethoxybenzene as an internal standard and HMF was not detected. ^a^ Isolated yield were obtained by column chromatography purification using hexane:ethyl acetate (2:1) as an eluent; n.d. = not detected.

**Table 3:** Textural properties of the studied SBA-16 samples.

Samples	S_BET_ (m^2^/g)	PD ^a^ (nm)	Pore Volume (cm^3^/g)
SBA-16	1128	7.90 (2.5)	0.64
CuSBA-16	695	7.55 (2.5)	0.50
MnSBA-16	630	7.86 (2.5)	0.39
VSBA-16	570	7.83 (2.5)	0.37

^a^ Pore diameter calculated by the Non-Local Density Functional Theory (NLDFT) method (adsorption branch).

**Table 4:** Oxidation of CMF for four consecutive flow reactions using CuSO_4_·Si 10%.

Consecutive Reactions	Yield (%)				
	DFF	CMF	HMF	OBMF	Total
1st	43 (20)	15 (16)	40 (43)	0 (20)	98 (98)
2nd	22	16	20	36	95
3rd	11	5	20	56	92
4th	17	n.d.	n.d.	77	94

*Reaction conditions for each flow experiment*: Each solution of CMF (225 mg, 1.56 mmol) and pyridine *N*-oxide (4 equiv.) in TGDE (0.5 M) was passed at a flow rate of 0.5 mL/min through the column totally filled with CuSO_4_·Si 10% at 160 °C; yields were determined by ^1^H-NMR analysis of the crude reaction mixture using 1,3,5-trimethoxybenzene as an internal standard; the results obtained for a fresh catalyst at 180 °C are provided in brackets; n.d. = not detected.

**Table 5:** Oxidation of CMF under flow conditions using different heterogeneous catalysts.

Entry	Catalyst	Flow Rate (mL/min)	Residence Time (min)	Yield (%)				
				DFF	CMF	HMF	OBMF	Total
1	CuSO_4_·Si 10% ^a,b^	0.5	5.3	40	16	42	n.d.	98
2	CuSO_4_·Si 10% dry ^a,c^	0.5	5.3	36	26	36	n.d.	98
3	CuSO_4_·Si 10% dry ^c^	0.5	5.3	40	20	25	10	94
4	CuSO_4_^d^	0.5	5.3	17	n.d.	71	n.d.	88
5	CuSO_4_·Si 5% ^c^	0.5	5.3	40	10	32	n.d.	82
6	Cu_2_O/silica (1:1) ^e^	0.5	5.3	37	n.d.	n.d.	n.d.	37
7	Cu-SBA16 ^a,f^	0.5	5.3	24	n.d.	58	n.d.	82
8	Cu/SiO_2_ (0.5 mol equiv.) ^c^	0.5	5.3	29	n.d.	49	n.d.	70
9	Cu/SiO_2_ (0.5 mol equiv.) ^c^	1.0	2.7	42	n.d.	13	18	73
10	Cu/SiO_2_ (1 mol equiv.) ^c^	1.0	2.7	54	n.d.	19	n.d.	72

*Reaction conditions for each flow experiment*: Each solution of CMF (225 mg, 1.56 mmol) and pyridine *N*-oxide (4 equiv.) in TEGDME (0.5 M) was passed at a specific flow rate through the column containing the heterogeneous Cu based catalyst at 160 °C; the yields were determined by ^1^H-NMR using 1,3,5-trimethoxybenzene as an internal standard; ^a^ TGDE: ^b^ amount of catalyst was 2.08 g; ^c^ column completely filled with catalyst; ^d^ column filled with 150 mg of silica and CuSO_4_ triturated (4.28 g); ^e^ column filled with 360 mg of silica and the rest was completely filled with catalyst (1:1 weight mixture of CuO_2_/Silica); ^f^ column filled with 1.05 g of silica and the remaining volume was completely filled with catalyst; n.d. = not detected.

## Supplementary Materials

Supplementary materials are available online.
