# Supplementary material for: Oxidation of 5-Chloromethylfurfural (CMF) to 2,5-Diformylfuran (DFF)
Source: Molecules. 2017 Feb 20;22(2):329. doi: 10.3390/molecules22020329 (PMC6155580; doi:10.3390/molecules22020329)
Supplement: Supplementary file 1 [file molecules-22-00329-s001.pdf]

Supplementary Material

Oxidation of 5-Chloromethylfurfural (CMF) to 2,5-diformylfuran (DFF)

Ana I. Vicente[1], Jaime A. S. Coelho[1], Svilen P. Simeonov[1,2], Hristina I. Lazarova[2], Margarita D. Popova[2] and Carlos A. M. Afonso[1,*]

[1] Research Institute for Medicines (iMed.ULisboa), Faculty of Pharmacy, Universidade de Lisboa, Av. Prof. Gama Pinto, 1649-003 Lisboa, Portugal. E-mail: carlosafonso@ff.ulisboa.pt

[2] Institute of Organic Chemistry with Centre of Phytochemistry Bulgarian Academy of Sciences, Acad. G. Bonchev str., bl. 9, 1113, Sofia, Bulgaria. E-mail: svilen.p.simeonov@gmail.com

* Correspondence: carlosafonso@ff.ulisboa.pt; Tel.: +351-217-946-400

Experimental

Materials

Tetrabutylammonium chloride (TBACl), 1,3,5-trimethoxybenzene, urea hydrogen peroxide (UHP), sodium perborate tetrahydrate ($NaBO_3.4H_2O$), calcium hypochlorite ($Ca(ClO)_2$), sodium periodate ($NaIO_4$), hydrogen peroxide (H_2O_2), oxone, potassium nitrite (KNO_2), *N*-methylmorpholine-*N*-oxide (NMO), potassium carbonate (K_2CO_3), potassium bromide (KBr), pyridine *N*-oxide (PNO), (2,2,6,6-tetramethylpiperidin-1-yl)oxyl (TEMPO), sodium nitrite ($NaNO_2$), *p*-tolyl sulfoxide, chlorotrimethylsilane, copper(I) chloride (CuCl), copper(II) triflate ($Cu(OTf)_2$), copper(I) triflate toluene complex (Cu(OTf)), copper oxide (Cu_2O) and copper(II) sulphate ($CuSO_4$) were all purchased from Sigma Aldrich. All these reagents were used as supplied without further purification. Chromatography column was performed with silica gel 60 GF254 MercK (Ref. 1.07730.1000). Catalysts were adsorbed or immobilized in silica gel 60 GF254 (Ref. 1.07730.1000), aluminium oxide 90 active basic (Ref. 101076) (AB) and aluminium oxide 90 active neutral (Ref. 101077) (AN) all from Merck. The ^{1}H-NMR spectra were recorded using $CDCl_3$ also purchased from Sigma Aldrich. The reagents dioctyl ether (Ref. 249599), tetraethylene glycol dimethyl ether (Ref. 172405) (TEGDME), triethylene glycol dimethyl ether (Ref. 8.08249.0250) (TGDE) were purchased from Sigma Aldrich and polyglycol 300 (PEG-6) was obtained from Clariant. All solvents, tetrahydrofuran (THF), acetonitrile, *tert*-butyl alcohol (*t*-BuOH), chloroform, ethyl acetate, dichloromethane and *n*-hexane were used without further purification.

Instrumentation

^{1}H-NMR spectra were obtained on Bruker spectrometer MX300 operating at 300 MHz. The microwave experiments were performed on a CEM Discover Benchmat apparatus using glass pressure vessel (10 mL).

Preparation of heterogeneous catalysts

General procedure for the adsorption of $CuSO_4$ in silica ($CuSO_4$.Si,) basic alumina ($CuSO_4$.AB) or neutral($CuSO_4$.AN) alumina

For the preparation of 10% (w/w) $CuSO_4$ adsorbed in silica, was dissolved the catalyst (1 g) in water (50 mL) and added the silica, basic or neutral alumina (10 g) and stirred for 10 minutes. The solvent was

evaporated to dryness in a rotaevaporator and left in an oven at 130 °C for 24h and used without further treatment.

General procedure for the preparation of the Cu/SiO_2 immobilized catalysts

The Cu/SiO_2 catalysts were prepared according the reported procedure.[1] Briefly, to prepare a catalyst with x%, to a solution of $Cu(NO_3)_2.2.5H_2O$ was added NH_4OH until pH 9. The silica was added and left stirring for 20 minutes. The solution was held in an ice bath at 0 °C and diluted in water in order to hydrolyze the copper complex and precipitation of the dispersed product. The solid was filtered and washed with water, dried overnight at 110 °C and calcined at 400 °C for 4h.

Synthesis of SBA-16 and its Mn-, Cu- and V-modified analogs

The parent silica SBA-16 material was synthesized according to the procedure of Hu *et al.*[2] Pluronic F127 triblock copolymer and cetyl trimethyl ammonium bromide (CTAB) were used as templates and tetraethylortosilicate (TEOS) as a silica source. In a typical synthesis, 1.0 g F127 and 0.12 g CTAB were completely dissolved into a solution of 130 ml water and 10 ml concentrated HCl, followed by the addition of 4.0 ml TEOS under stirring. After 1 h of stirring at 40 _C, the mixture was heated at 80 oC for 24 h under static conditions. The solid product was filtrated, washed with water three times and dried at 50 ^oC. The templatewas removed by calcination in air at 550 ^oC for 5 h with a heating rate of 1 ^oC/min.
Mn, Cu, V-containing (5-20 wt %) SBA-16 materials were prepared by incipient wetness impregnation with manganese (II) acetylacetonate, copper (II) acetylacetonate and vanadyl(VI) sulphate as salts precursors, respectively.

Characterization of the initial and modified SBA-16 materials: The X-ray powder diffraction (XRD) patterns were recorded on a PANalytical X´Pert PRO (HTK) high-resolution diffractometer using Cu Kα1 radiation (1.5406 Å) in the 2 θ range from 5° to 80° (100 s per step 0.016°) for the samples and from 10° to 70° (100 s per step 0.016°) for the sample holder using a fully opened X´Celerator detector.

Nitrogen physisorption measurements were carried out at -196°C using Tristar 3000 Micromeritics volumetric adsorption analyzer. Before the adsorption analysis, the samples were outgassed under vacuum for 2 h at 200°C in the port of the adsorption analyzer. The BET specific surface area was calculated from adsorption data in the relative pressure range from 0.05 to 0.21 The pore size distributions (PSDs) were calculated from nitrogen adsorption data using an algorithm based on the ideas of Barrett, Joyner, and Halenda (BJH).

The reducibility of Cu, Mn and Vmodified samples were investigated by temperature-programmed reduction (TPR) technique in H_2/Ar flow (10:90, 20 ml/min) using a conventional TPR apparatus equipped with a heat conductivity cell and a trap for removal of released water. Before TPR run samples were pretreated in oxygen at 350 ^oC for 1h.

Preparation of 5-(hydroxymethyl)furfural (HMF)

HMF was synthetized from fructose using the protocol reported previously by us, using tetraethylammonium bromide/water as reaction medium and Amberlyst-15 as catalyst.[3] ^{1}H-NMR (300 MHz, $CDCl_3$) δ 2.59 (s, 1H), 4.71 (s, 2H), 6.51 (d, *J* = 3.5 Hz, 1H), 7.21 (d, *J* = 3.5 Hz, 1H), 9.58 (s, 1H).

Preparation of 5-(chloromethyl)furan-2-carbaldehyde (CMF)[4]

To a solution of HMF (2 g, 15.9 mmol) in dry CH_3Cl (50 mL) at 40 °C, chlorotrimethylsilane (10 g, 0.11 mol) was slowly added and the reaction was left reacting overnight. The reaction was washed with $NaHCO_3$ and extracted with dichloromethane. The solvent was evaporated and the crude was purified by chromatographic column with hexane/ethyl acetate 2:1 as eluent to obtain 1.56 g of a yellow oil (η=68 %). ^{1}H RMN (300 MHz, $CDCl_3$) δ 4.55 (s, 2H), 6.53 (d, *J*=3.6 Hz, 1H), 7.15 (d, *J*=3.3 Hz, 1H), 9.56 (s, 1H).

Procedure for the oxidation of CMF in TBACl and using UHP, $NaBO_3.4H_2O$, $Ca(ClO)_2$, $NaIO_4$, H_2O_2, oxone, KNO_2 or NMO as oxidants

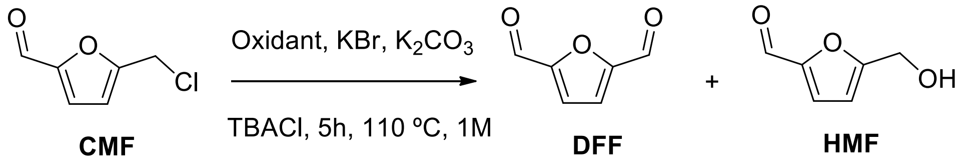

In a glass pressure tube (Aldrich Ref. Z181064-1EA) was added 5-chloromethylfurfural (CMF) (50 mg, 0.35 mmol), 1.5 equivalents of an oxidant, 2 eq. of K_2CO_3, 0.3 eq. of KBr and tetrabutylamonnium chloride (TBACl) (1 M) and left stirring for 5 hours at 110 °C. For the reactions performed with UHP and NMO was not added K_2CO_3 and for the reaction with oxone the concentration was of 0.5 M since for higher concentrations the solution become saturated. The solution was filtered in silica using a mixture of hexane/ethyl acetate (1:1 and the solvents were evaporated. Yields of CMF, 2,5-diformylfuran (DFF) and 5-hydroxymethylfurural (HMF) were determined by ^{1}H-NMR using 1,3,5-trimethoxybenzene as internal standard (Table S1).

Procedure for the oxidation of CMF using pyridine *N*-oxide in TBACl

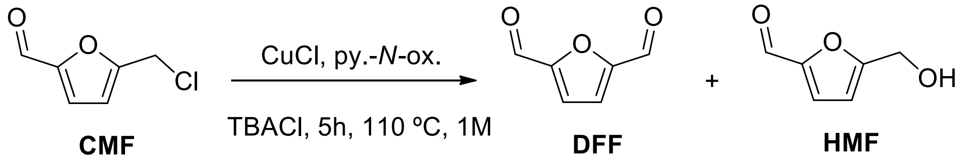

In a glass pressure tube (Aldrich Ref. Z181064-1EA) was added CMF (50 mg, 0.35 mmol), 4 eq. of pyridine *N*-oxide and 0.5 eq. of CuCl in tetrabutylamonnium chloride (TBACl) (1 M) and left stirring for 5 hours at 110 °C. The solution was filtered in silica using a mixture of hexane/ethyl acetate 1:1 and the solvents

were evaporated. Yields of CMF, 2,5-diformylfuran (DFF) and 5-hydroxymethylfurural (HMF) were determined by ^{1}H-NMR using 1,3,5-trimethoxybenzene as internal standard. (Table S1).

Table S1. Yields of DFF and HMF for the oxidation reactions of CMF performed in tetrabutylamonnium chloride.

Oxidant	Yield (%)	
	DFF	HMF
UHP[a, c]	-	-
$NaBO_3.4H_2O$	1	3
$Ca(ClO)_2$	1	17
$NaIO_4$	1	21
H_2O_2	3	17
Oxone[b]	4	45
KNO_2	7	26
NMO [c]	9	18
Py. *N*-ox.[c, d]	12	67
Py. *N*-ox.[c, d, e]	8	2
Py. *N*-ox.[c, d, f]	12	52

Reaction conditions: CMF (50 mg, 0.35 mmol), oxidant (1.5 eq.) , K_2CO_3 (2 eq.) and of KBr (0.3 eq.) in TBACl (1 M) at 110 °C in a glass pressure tube (Aldrich Ref. Z181064-1EA); yields were determined by ^{1}H-NMR using 1,3,5-trimethoxybenzene as internal standard and no CMF was detected; [a] formation of a solid; [b]c=0.5M; [c]0 eq. K_2CO_3; [d]0.5 eq. CuCl and 4 eq. of py. *N*-ox.; [e]dry py. *N*-ox. and TBACl for 5h; [f] T=160 °C.

Attempt for the oxidation of CMF using TEMPO as oxidant

In a glass pressure tube (Aldrich Ref. Z181064-1EA) was added CMF (50 mg, 0.35 mmol), 3 mol% equivalents of 2,2,6,6-tetramethylpiperidin-1-yl)oxyl (TEMPO), 12.5 mol% eq. of KBr and 6.3 mol% eq. of $NaNO_2$ in acetonitrile (CH_3CN) or *tert*-butyl alcohol (*t*-BuOH) at 80 ˚C for 3 days. Following the reaction by thin layer chromatography, was not observed the formation of new products.

Attempt for the oxidation of CMF using *p*-tolyl sulfoxide

In a glass pressure tube (Aldrich Ref. Z181064-1EA) was added CMF (50 mg, 0.35 mmol), 0.3 %mol eq. of KBr and 10 eq. of *p*-tolyl sulfoxide at 100 ˚C. After 24 hours of reaction the solid was dissolved in hot MeOH and left crystalizing. When some crystals were formed, the solution was filtered and washed with cold MeOH and the solution was concentrated and analysed by ^{1}H-NMR. This reaction was also performed at 150 ˚C. The ^{1}H NMR spectra of the reactions showed no signals of CMF, DFF or HMF.

General procedure for the oxidation of CMF using pyridine *N*-oxide as oxidant

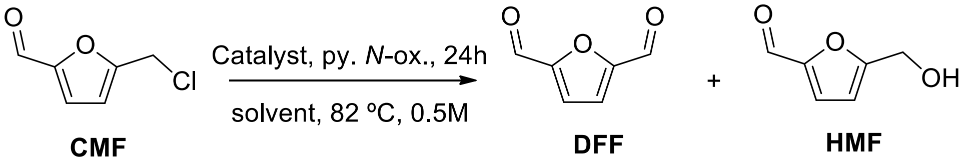

In a glass pressure tube (Aldrich Ref. Z181064-1EA) was added CMF (50 mg, 0.35mmol), 2 eq. of pyridine *N*-oxide, 0.5 eq. of CuCl and CH_3CN (0.5 M) and left stirring for 24 hours at 82 °C. The solution was filtered in silica using a mixture of hexane/ethyl acetate 1:1 and the solvents were evaporated. Yields of CMF, 2,5-diformylfuran (DFF) and 5-hydroxymethylfurural (HMF) were determined by ^{1}H-NMR using 1,3,5-trimethoxybenzene as the internal standard. The optimization of this reaction consisted in the variation of parameters such as solvent, temperature, concentration of oxidant or reaction.

Table S2. Reaction conditions and yields of DFF, CMF and HMF for the oxidation reactions of CMF performed using pyridine *N*-oxide as oxidant under thermal heating.

Solvent	Catalyst (eq.)	Oxidant (eq.)	T °C	Yield (%)			
				DFF	CMF	HMF	Total
CH_3CN	CuCl	2	82	9	49	n.d.	58
t-BuOH	CuCl	2	82	5	47	48	100
THF	CuCl	2	82	6	n.d.	96	100
CH_3CN	None	2	82	n.d.	100	n.d.	100
CH_3CN[a]	CuCl	2	82	n.d.	100	n.d.	100
CH_3CN:H_2O 1:1	CuCl	2	82	n.d.	n.d.	100	100
CH_3CN[b]	CuCl	2	82	15	2	23	40
CH_3CN	$Cu(OTf)_2$	2	82	13	25	n.d.	38
CH_3CN	CuCl	4	82	37	3	26	66
CH_3CN[c]	CuCl	4	82	25	3	53	81
CH_3CN[d]	CuCl	4	82	24	3	34	61
CH_3CN[e]	CuCl	4	82	34	14	n.d.	48
CH_3CN	CuCl	8	82	37	8	4	49
CH_3CN	CuCl	4	60	13	2	45	60
CH_3CN	CuCl	4	110	19	n.d.	n.d.	19
CH_3CN[f]	CuCl	4	110	n.d.	n.d.	n.d.	--
CH_3CN	CuOTf	4	82	31	2	3	36
CH_3CN	$Cu(OTf)_2$	4	82	35	5	n.d.	40
CH_3CN[g]	$Cu(OTf)_2$	4	82	32	2	4	38
CH_3CN[h]	CuCl	4	82	n.d.	100	n.d.	100

Reaction conditions: CMF (50 mg, 0.35 mmol), catalyst (0.5 eq.), pyridine *N*-oxide as oxidant, in a concentration of 0.5 M at 82 °C for 24h in a glass pressure tube (Aldrich Ref. Z181064-1EA); yields were determined by ^{1}H-NMR using 1,3,5-trimethoxybenzene as internal standard. [a] c=0.1M; [b] 0.3 eq. KBr; [c] dry py. *N*-ox. and CH_3CN; [d] dry py. *N*-ox. and CH_3CN and 0.3 eq. of KBr; [e] 55 h of reaction; [f] extration with HCl 1M; [g] 0.25 eq. of catalyst; [h] Reaction performed using HMF (0.35 mmol) instead of CMF; n.d.=not detected.

General procedure for the oxidation of CMF under microwave irradiation (MWI)

In a microwave pressure vessel (10 mL) was added 1 equivalent of 5-chloromethylfurfural (CMF), (50 mg, 0.35 mmol), 4 eq. of pyridine *N*-oxide, 0.5 eq. of $Cu(OTf)_2$ and CH_3CN (0.5 M) and left stirring for different times and temperatures. The solution was filtered in silica using a mixture of hexane/ethyl acetate 1:1 and the solvents were evaporated. Yields of CMF, 2,5-diformylfuran (DFF) and 5-hydroxymethylfurural (HMF) were determined by ^{1}H-NMR using 1,3,5-trimethoxybenzene as the internal standard. The isolated yield for DFF and CMF was obtained by column chromatography purification using hexane:ethyl acetate (2:1) as eluent. ^{1}H-NMR (300 MHz, $CDCl_3$) 7.27 (s, 2H), 9.79 (s, 2H).

Table S3. Reaction conditions and yields of DFF, CMF and HMF for the oxidation reactions of CMF performed under microwave irradiation (MWI).

Time	Temp. (°C)	Oxidant (eq.)	Catalyst (eq.)	Yield (%)	
				DFF	CMF
5h[a)]	82	4	0.5	24	38
6h	82	4	0.5	49	24
12h	82	4	0.5	32	19
1h	100	4	0.5	24	5
2h	100	4	0.5	39	4
3h	100	4	0.5	36	10
1h	120	4	0.5	39	7
1h	120	2	0.5	29	36
1h	120	4	0.25	14	28
1h30	120	2	0.5	33	16
5 min	140	4	0.5	44	5
15 min	140	4	0.5	46	5
30 min	140	4	0.5	47	6
1h	140	4	0.5	49	4
1 min	160	4	0.5	47	18
**5 min**	**160**	**4**	**0.5**	**51**	**8**
5 min[b)]	160	4	0.5	50	8
5 min	160	4	0	6	59
5 min	160	4	0.25	39	8
5 min	160	4	1	37	8
5 min	160	2	0.5	40	n.d.
5 min	160	0	0.5	dark crude	
5 min	160	4	0.4	46	9
5 min[c)]	160	2	0.5	9	7
30 min	160	4	0.5	45	4
1 min	170	4	0.5	49	3

10 min	170	4	0.5	36	5
1 min	180	4	0.5	37	1

Reaction conditions: CMF (50 mg, 0.35 mmol), $Cu(OTf)_2$ as catalyst, pyridine *N*-oxide as oxidant, in CH_3CN (0.5 M) under microwave irradiation (MWI); yields were determined by ^{1}H-NMR using 1,3,5-trimethoxybenzene as internal standard and HMF was not detected; [a)] the reaction was interrupted at 20 min, 1h and 3h for TLC analysis; [b)] isolated yield; [c)] 2 eq. of NMO; n.d.=not detected.

Table S4. Observed yields of DFF, CMF and HMF for the oxidation reactions performed using several catalysts under microwave irradiation.

Time (min.)	Catalyst (mol% eq.)	Yield (%)		
		DFF	CMF	HMF
5	$Cu(OTf)_2$ (0.5)	51	8	n.d.
5	CuCl (0.5)	35	11	n.d.
5	CuSBA-16 10% (0.5)	50	n.d.	n.d.
5	$CuSO_4$ (0.5)	42	31	n.d.
10	$CuSO_4$ (0.5)	38	29	n.d.
10[a]	$CuSO_4$ (0.5)	35	5	n.d.
30	$CuSO_4$ (0.5)	41	4	n.d.
5	$CuSO_4$ (1)	38	n.d.	7
10	$CuSO_4$ (1)	33	14	n.d.
5	$CuSO_4$.Si 10% (0.5)	31	12	1
5	$CuSO_4$.Si 5% (0.5)	10	4	2
5	$CuSO_4$.AN 10% (0.5)	21	n.d.	4
5	$CuSO_4$.AB 10% (0.5)	26	n.d.	2
5	10% MnSBA-16, 10% VSBA-16[b]	--	--	--

Reaction conditions: CMF (50 mg, 0.35 mmol), catalyst, pyridine *N*-oxide (4 eq.), in CH_3CN (0.5 M) at 160 °C under microwave irradiation; yields were determined by ^{1}H-NMR using 1,3,5-trimethoxybenzene as internal standard; [a] 170 °C; [b] the temperature was not stable reaching 240 °C and the reaction vessel broken; n.d.=not detected, AN= aluminium oxide neutral, AB= aluminium oxide basic.

Table S5. Observed yields of DFF and CMF using different solvents under microwave irradiation

Solvent	Catalyst	Yield (%)	
		DFF	CMF
CH_3CN	$Cu(OTf)_2$	51	8
CH_3CN[a]	$Cu(OTf)_2$	9	7
THF	$Cu(OTf)_2$	51	17
dioctyl ether	$Cu(OTf)_2$	24	5
PEG-6	$Cu(OTf)_2$	23	n.d.
TGDE	$Cu(OTf)_2$	54	16
TGDE	$CuSO_4$	33	0
TGDE	$CuSO_4$.Si 10%	44	22

Reaction conditions: CMF (50 mg, 0.35 mmol), 0.5 eq. of catalyst, pyridine *N*-oxide (4 eq.), in a solvent at 0.5 M at 160 °C for 5 minutes under microwave irradiation; yields were determined by ^{1}H-NMR using 1,3,5-trimethoxybenzene as internal standard; no HMF was found;; [a] 2eq. of NMO (*N*-methylmorpholine-*N*-oxide); TGDE=Triethylene glycol dimethyl ether; n.d.=not detected

Procedure for the oxidation of CMF by catalyst reuse

In a microwave pressure vessel (10 mL) was added 5-chloromethylfurfural (CMF), (50 mg, 0.35 mmol), 4 eq. of pyridine *N*-oxide, 0.5 eq. of $CuSO_4$.Si (10% w/w) and TGDE (0.5 M) and left stirring for 5 minutes at 160 °C under microwave irradiation. The solution was filtered using a Pasteur pipette with cotton to separate the silica from the reaction. The crude was filtered in silica using a mixture of hexane/ethyl acetate 1:1 and the solvents were evaporated. Yields of CMF, DFF, HMF and (5,5'-(oxybis(methylene))bis(furan-5,2-diyl))dimethanol (OBMF) were determined by ^{1}H-NMR using 1,3,5-trimethoxybenzene as the internal standard. The catalyst was reused for the next cycle and the microwave flask filled with the same proportions of reagents used in the first one.

Table S6. Catalyst recycling experiments.

Cycle	Yield (%)				
	**DFF**	**CMF**	**HMF**	**OBMF**	**Total**
1st	43	13	n.d.	n.d.	56
2nd	40	12	6	10	68
3rd	37	36	4	22	98
4th	46	29	n.d.	n.d.	74
5th	33	47	4	6	90
6th	38	38	n.d.	n.d.	76
7th	38	57	n.d.	n.d.	95

Reaction conditions: CMF (50 mg, 0.35 mmol), $CuSO_4$.Si 10% (0.5 eq), pyridine *N*-oxide (4 eq.) in TGDE (0.5 M) at 160 °C for 5 minutes under microwave irradiation; yields were determined by ^{1}H NMR using 1,3,5-trimethoxybenzene as internal standard; n.d.=not determined.

General procedure for the oxidation of CMF using flow conditions

An empty HPLC column (i.d. = 3.9 mm; L= 300 mm) was filled with the respective catalyst, and placed inside a gas chromatographer (GC) oven. The solvent was passed through the column at room temperature using an HPLC pump, after the column was completely filled with the solvent, the temperature of the GC oven was raised to the desired value and left 30 minutes to stabilize and then the solution containing CMF (225 mg, 1.56 mmol) and pyridine *N*-oxide (4 eq.) in the solvent (0.5 M of CMF) was passed through the column at the constant flow and collected in different vials. The ^{1}H NMR quantification of each product of the collected solutions was performed using a specific weight of the sample and adding a specific amount of the internal standard, 1,3,5-trimethoxybenzene. The weigh

percentage of catalyst to be used in the flow reactions was calculated by weighing the column before and after filling with the catalyst. If necessary, the resulting empty volume of the column was filled with silica.

Table S7. Observed yields of DFF, CMF, HMF and OBMF for consecutive flow reactions using $CuSO_4$.Si 10%.

Consecutive Reactions	Yield (%)				
	DFF	CMF	HMF	OBMF	Total
1st	43 (20)	15 (16)	40 (43)	0 (20)	98 (98)
2nd	22	16	20	36	95
3rd	11	5	20	56	92
4th	17	n.d.	n.d.	77	94

Reaction conditions for each flow experiment: Each solution of CMF (225 mg, 1.56 mmol) and pyridine *N*-oxide (4 eq.) in TGDE (0.5 M) was passed at a flow rate of 0.5 mL/min through the column totally filled with $CuSO_4$.Si 10% at 160 °C; yields were determined by ^{1}H-NMR using 1,3,5-trimethoxybenzene as internal standard; in brackets is provided the results obtained for a fresh catalyst at 180 °C; n.d.=not detected.

Table S8. Observed yields of DFF, CMF, HMF and OBMF under flow conditions using different heterogeneous catalysts.

Catalyst	Yield (%)				
	DFF	CMF	HMF	OBMF	Total
$CuSO_4$.Si 10%[a,b]	40	16	42	n.d.	98
$CuSO_4$.Si 10% dry[a,c]	36	26	36	n.d.	98
$CuSO_4$.Si 10% dry[c]	40	20	25	10	94
$CuSO_4$[d]	17	n.d.	71	n.d.	88
$CuSO_4$.Si 5%[c]	40	10	32	n.d.	82
Cu_2O/silica (1:1)[e]	37	n.d.	n.d.	n.d.	37
Cu/SiO_2 (0.5 mol eq.)[c]	29	n.d.	49	n.d.	70
Cu-SVA16[a,f]	24	n.d.	58	n.d.	82

Reaction conditions for each flow experiment: Each solution of CMF (225 mg, 1.56 mmol) and pyridine *N*-oxide (4 eq.) in TEGDME (0.5 M) was passed at a flow rate of 0.5 mL/min through the column containing the heterogeneous Cu based catalyst at 160 °C; yields were determined by ^{1}H-NMR using 1,3,5-trimethoxybenzene as internal standard; [a] TGDE, [b] amount of catalyst was 2.08 g, [c] column completely filled with catalyst, [d] column filled with 150 mg of silica and $CuSO_4$ triturated (4.28 g), [e] column filled with 360 mg of silica and the rest was completely filled with catalyst (1:1 weight mixture of CuO_2/Silica), [f] column filled with 1.05 g of silica and the rest was completely filled with catalyst; n.d.=not detected.

Table S9. Observed yields for DFF, CMF, HMF and OBMF for different flow rates.

**Flow rate** (mL/min)	**Catalyst**	**Yield (%)**				
		**DFF**	**CMF**	**HMF**	**OBMF**	**Total**
0.5	Cu/SiO_2 (0.5 mol eq.)	29	n.d.	49	n.d.	70
1	Cu/SiO_2 (0.5 mol eq.)	42	n.d.	13	18	73
1	Cu/SiO_2 (1 mol eq.)	54	n.d.	19	n.d.	72
1.5	Cu/SiO_2 (1 mol eq.)	53	n.d.	n.d.	n.d.	53

Reaction conditions for each flow experiment: Each solution of CMF (225 mg, 1.56 mmol) and pyridine *N*-oxide (4 eq.) in TEGDME (0.5 M) was passed at a specific flow rate through the column totally filled with Cu/SiO_2 at 160 °C; yields were determined by ^{1}H-NMR using 1,3,5-trimethoxybenzene as internal standard; n.d.=not detected.

Table S10. Yields for the continuous flow reaction

**Fraction**	**Yield (%)**	
	**DFF**	**HMF**
1st	8	1
2nd	7	n.d.
3rd	2	7
4th	3	11
5th	2	12
6th	3	14
7th	2	9
Total	27	54

Reaction conditions for the flow experiment: Solution of CMF (1 g, 7.02 mmol) and pyridine *N*-oxide (4 eq.) in TEGDME (0.5 M) was passed at a flow rate of 0.5 mL/min through the column totally filled with Cu/SiO_2 (0.5 mol eq.) at 160 °C; Sequentially samples with same volume were collected and yields were determined by ^{1}H-NMR using 1,3,5-trimethoxybenzene as internal standard; no CMF was detected; n.d.= not detected.

^{1}H NMR spectra

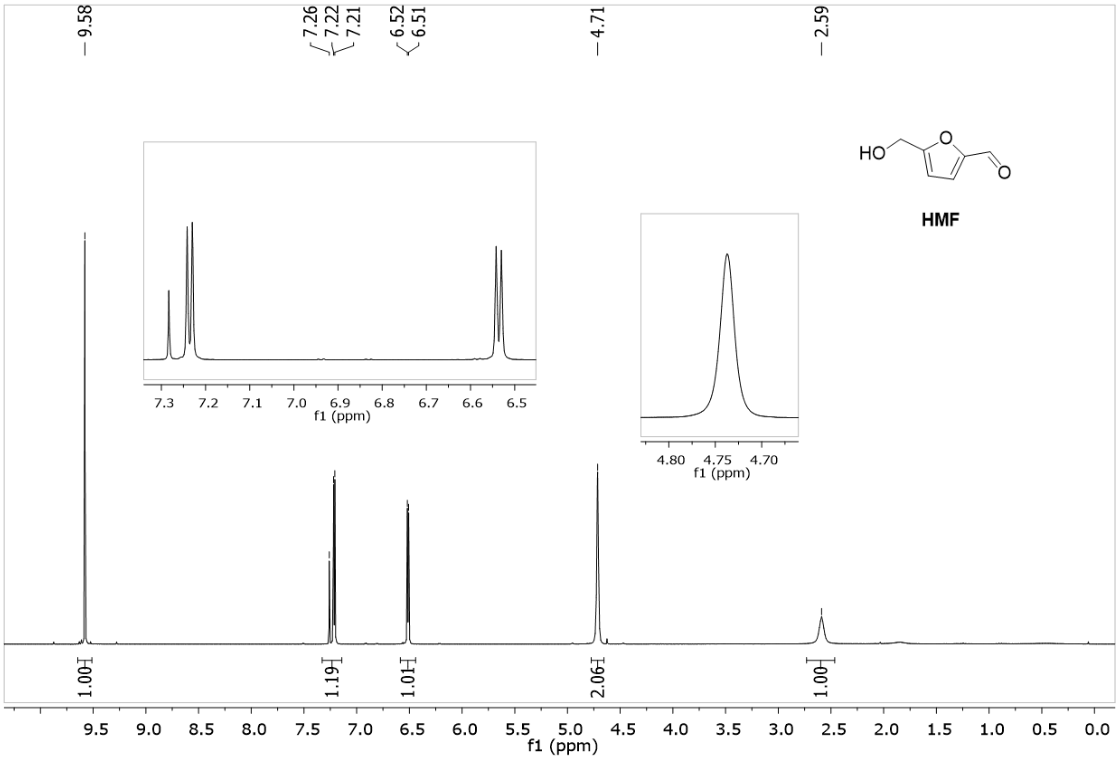

Figura S1. ^{1}H NMR spectrum of the HMF.

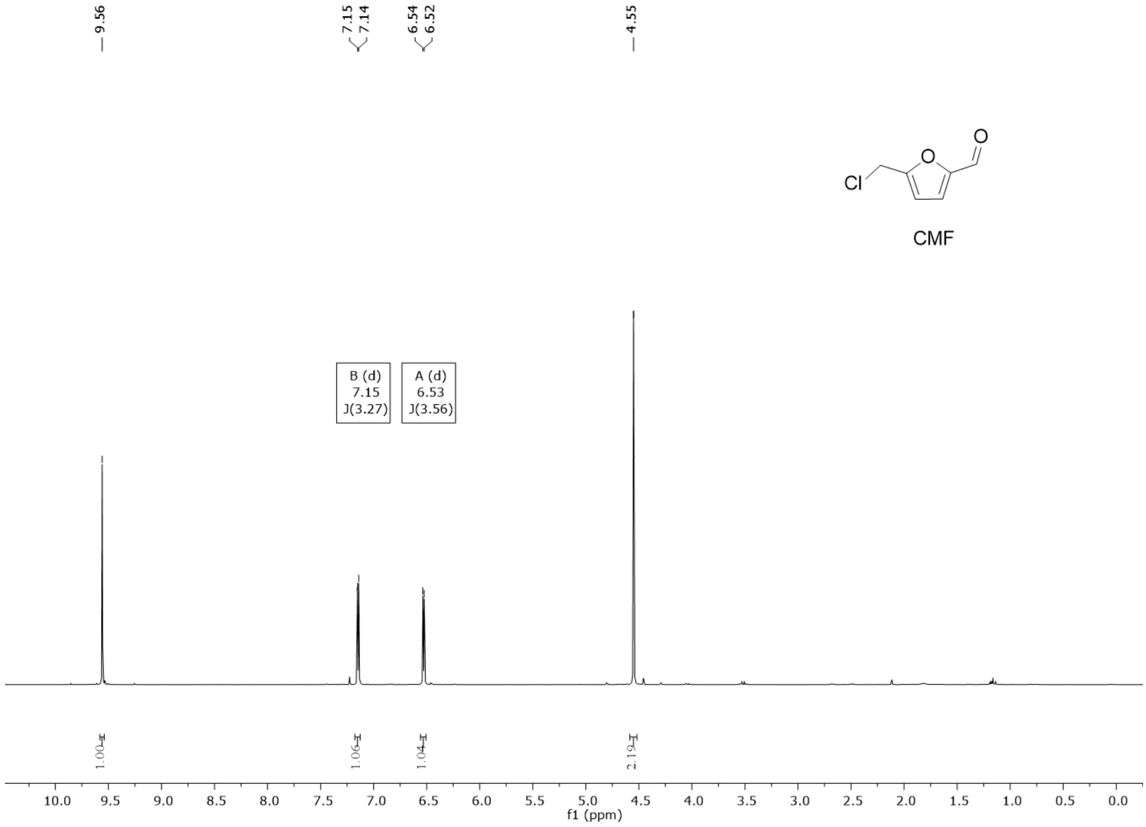

Figure S2. ^{1}H NMR spectrum of the CMF.

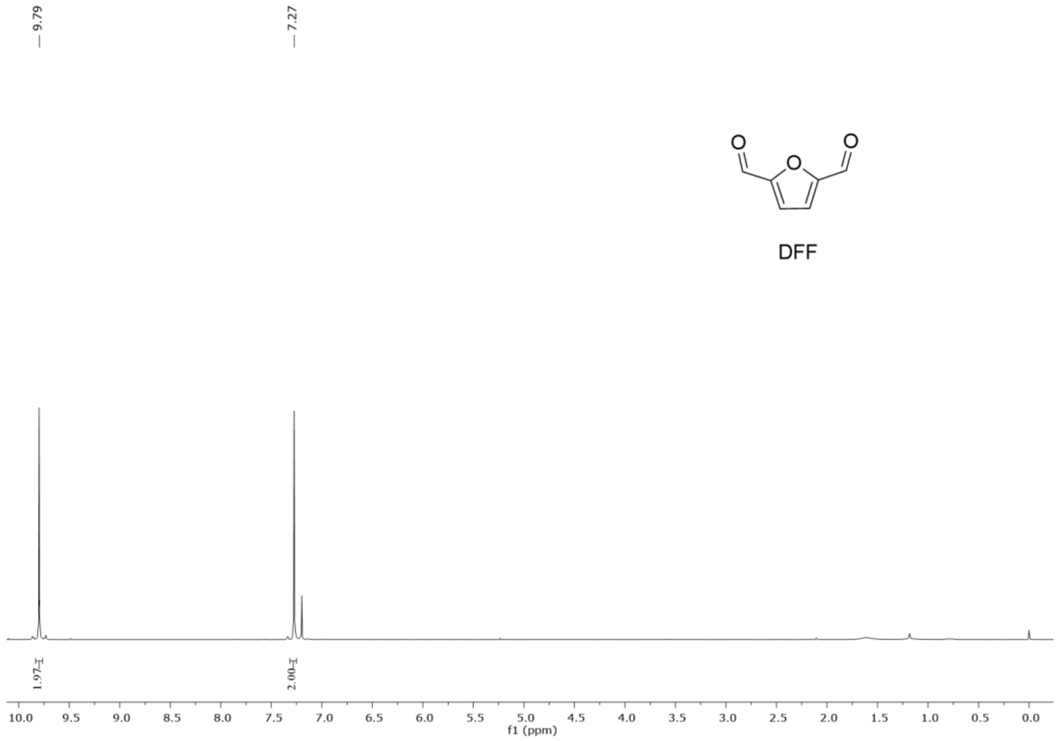

Figure S3. ^{1}H NMR spectrum of the DFF.

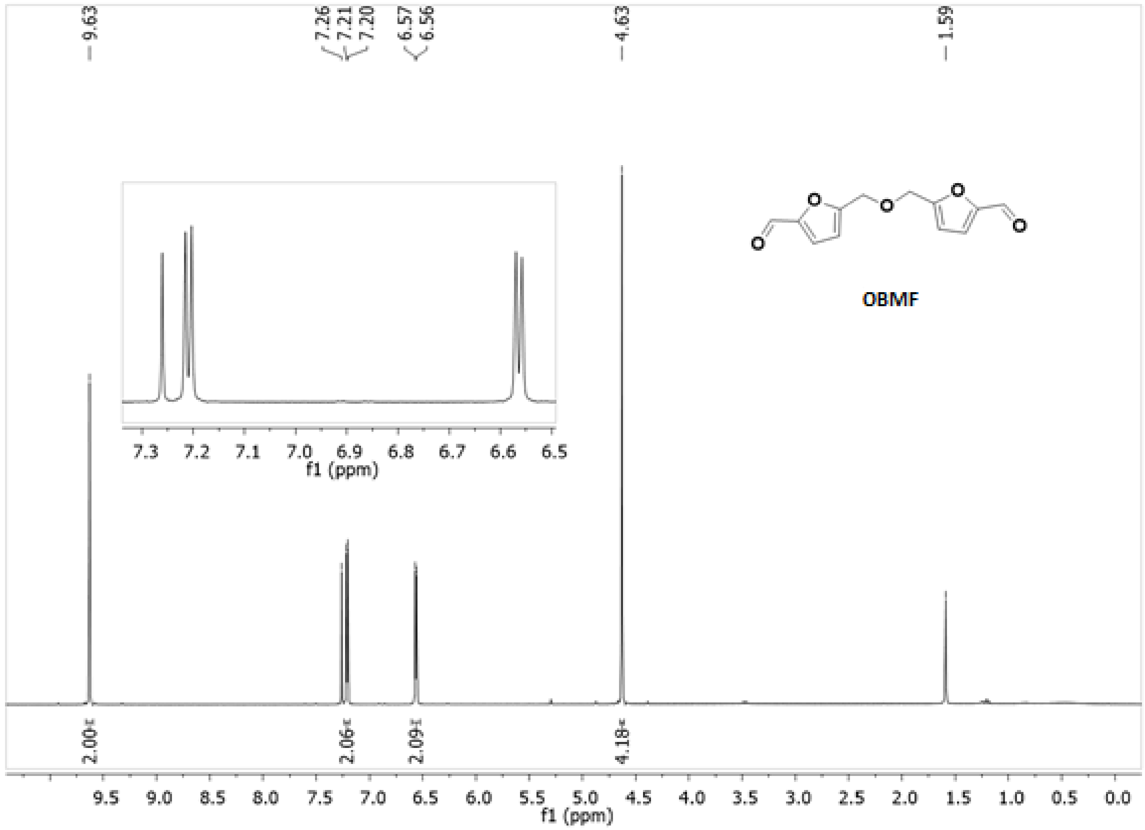

Figure S4. ^{1}H NMR spectrum of the OBMF.

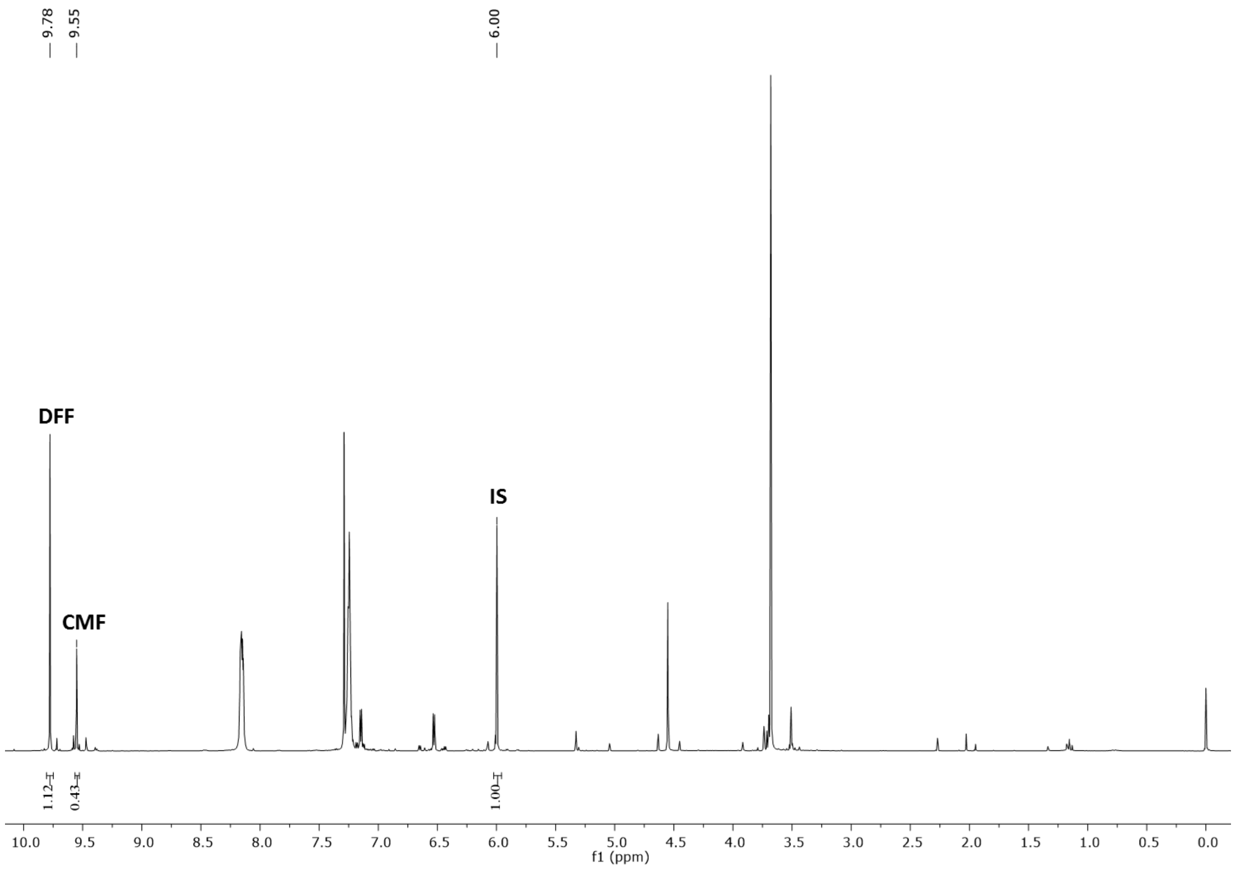

Figure S5. ^{1}H NMR spectrum of the crude after filtration obtained for the reaction performed in a reactor with 0.5 eq. of CuCl, 4 eq. of pyridine *N*-oxide, in a solution of 0.5 M in CH_3CN, at 82 °C for 24h. IS = Internal standard (1,3,5-trimethoxybenzene).

---

[1] P. Carniti, L. A. Gervasini, V. H. Modica, N. Ravasio, *Applied Catalysis B: Environmental,* **2000,** *28,* 175.
[2] Y. Hu, Zh Zhi, Q. Zhao, Ch Wu, P. Zhao, H. Jiang, T. Jiang, S. Wang, *Micropor. Mesopor. Mater.,* **2012,** *147,* 94.
[3] S. P. Simeonov, J. A. S. Coelho , C. A. M. Afonso *ChemSusChem*, 2012, **5**, 1388; Jaime A. S. Coelho, Svilen P. Simeonov, Carlos A. M. Afonso, *Org. Synth.*, 2016, **93**, 29.
[4] K. Sanda, L. Rigal, A. Gaset, *Cabohydrate Research,* **1989,** *187,* 15-23.
